# The Experience of Project-Based Learning Among First-Year Health Sciences Students in Morocco

**DOI:** 10.7759/cureus.72649

**Published:** 2024-10-29

**Authors:** Mohamed Taiebine, Wafaa Al Hassani, Chakib Nejjari

**Affiliations:** 1 Euromed Research Center, Euromed University of Fez, Fez, MAR

**Keywords:** course, education, health, learning, morocco, nursing, project

## Abstract

Introduction: Project-based learning (PBL) has emerged as a promising pedagogy for healthcare education. This study explored the implementation of PBL in undergraduate nursing and health sciences programs, focusing on the psychology and sociology of health courses.

Methods: PBL methodology was employed within the course, incorporating both face-to-face and asynchronous online sessions facilitated by Moodle® (Moodle Pty Ltd, Barcelona, Spain). Following the PBL activities, a postquestionnaire was used to assess student learning outcomes.

Results: The study found that the PBL approach, centered on group projects, fostered the development of essential teamwork, communication, and critical thinking skills required by future healthcare professionals. Additionally, PBL contributes to a dynamic and engaging learning environment, enhancing students' understanding and hard skills while simultaneously developing soft, self-, and life skills crucial for navigating the complexities of nursing practice.

Discussion: These findings suggest that PBL offers a valuable approach for preparing future healthcare professionals by equipping them with both technical knowledge and essential workplace skills.

## Introduction

Definition of PBL

Project-based learning (PBL) is a highly effective and innovative educational approach that places students at the center of their learning experience [1]. Unlike conventional methods that rely heavily on lectures and rote memorization, PBL emphasizes active engagement, critical thinking, and real-world application [2]. In a PBL setting, students tackle authentic, complex problems or challenges, often in an interdisciplinary manner. These projects mirror the types of situations that professionals encounter in their respective fields [3]. Furthermore, PBL in higher education offers several benefits, such as developing critical thinking and problem-solving skills, teamwork, engagement, and enhanced motivation [4].

PBL comprises five stages: preparation, implementation, presentation, evaluation, and revision. However, conventional PBL has been criticized for lacking creative thinking and design processes when applied to an application domain. To address this limitation, Hwang et al. [5] introduced an innovative PBL (iPBL) framework that integrates creative learning and the conceive-design-implement-operate (CDIO) frameworks. Creative learning enhances problem-solving creativity, while the CDIO provides a structured design process that leads to convergent results. The iPBL framework consists of seven stages: preparation, conception, design, implementation, operation, evaluation, and revision.

Importance of PBL in health sciences and nursing

Portfolio training and clinical evaluation methods have been demonstrated to improve the clinical competence of nursing students [6,7]. Portfolio-based education has been shown to significantly enhance students' clinical competence in cognitive, affective, and psychomotor domains. Moreover, portfolio training and clinical evaluation methods have been found to increase student satisfaction with the evaluation process, leading to higher engagement and participation in the learning process [8]. Tsai et al. [9] examined the employment of electronic portfolios among 15 novice nurses with less than two years of occupational experience. Their investigation revealed that e-portfolios are utilized in clinical settings, and a well-structured educational information system can cater to the requirements of nurses and expedite their learning advancement.

The PBL methodology consists of several stages, including anchoring, driving questions, investigation and research, creation and development, and presentation of results [10]. The anchoring phase introduces the context and problem to be addressed through various means, such as short narratives, data, images, videos, and news. The driving question phase then guides the focus of the project, which can be established by the tutor or jointly identified by the students. Following this, students conduct an in-depth investigation of the problem and then think strategically and collaboratively to create and develop solutions. Finally, the project is presented systematically through various strategies like oral presentations, dialogue posters, and videos. The effectiveness of this methodology depends on the level of engagement around the anchoring and driving question stages [10].

Implementing PBL in nursing education is paramount, as it enhances the learning experience, fosters critical thinking skills [11], and prepares students for the complex and dynamic healthcare environment. PBL allows students to engage in projects that mirror real-world healthcare scenarios, bridging the gap between theoretical knowledge gained in the classroom and the practical skills needed in clinical settings. It also promotes the development of interpersonal and intrapersonal communication skills while presenting findings and collaborating with peers. These experiences improve communication skills, both in written and verbal formats, which are vital for interacting with patients and their families. Such soft, life, and power skills prepare students to communicate and collaborate effectively with colleagues from different healthcare disciplines in the future.

When conducting literature and field research, the application of evidence-based research and practice is crucial in researching and staying informed about evidence-based information to solve sociocognitive bias and health misbeliefs [12,13]. In terms of the psychomotivational aspects of PBL, using such an approach may develop initiative, intellectual curiosity, self-motivation, adaptability, resilience [14], and coping strategies while exposing students to various scenarios, dilemmas, and vignettes as they navigate complex healthcare issues. Because learning is a lifelong and continuous process [15], PBL encourages students to stay updated and informed about new research, technologies, and practices in their field. By combining hard, soft, and life skills [16], PBL prepares students in nursing and health sciences for the complexities and demands of their future roles as healthcare professionals [17].

## Materials and methods

The course was initially taught to a total number of 57 first-year health sciences students enrolled in the Euromed University of Fez, Fez, Morocco. Participants were predominantly between 17 and 21 years of age, with a distribution of 45 women (78.9%) and 12 men (21.1%). PBL has been implemented between October and December 2023. The course, developed by the professor and the faculty of health sciences deanship, emphasized active student participation in following the curriculum and the proposal of PBL. The course design was collaborative, focusing on learning and exchanging experiences. We used convenience sampling, given that the participants were drawn from a group of students registered in the course.

On the one hand, during the first semester of 2023, deans and faculty members attended pedagogical meetings to evaluate and reassess implementation strategies. The first step was training professors in the methodology, which included reading materials and discussions supervised by experts.

On the other hand, students initially attended a theoretical session that introduced them to the learning methodology. Thereafter, they were segregated into eight groups based on the learning style inventory, which classifies them into four learning styles: diverging, assimilating, converging, and accommodating [18]. Upon completing the course, each of the selected groups showcased their projects. Table 1 provides a detailed account of the themes, educational objectives, instructional design, and final products of these projects.

**Table 1:** Titles of the projects that have been presented by each group HCWs: healthcare workers

Tutorial group	Driving question	Project theme	Educational purpose	Final product
Group 1	What is the role of nurses in euthanasia?	Euthanasia or medically assisted death	To understand the ethics, regulations, and motivations for euthanasia	Oral presentation
Group 2	What are the benefits and risks of traditional healers? How this practice may impact nursing?	Nursing and traditional healing	To investigate the beliefs and social perceptions of traditional healing in the Moroccan context	Oral presentation/video/social media dissemination
Group 3	What is the impact of new technology and artificial intelligence on nursing?	Nursing practice in the digital era	To explore the applications of new technologies in nursing and how artificial intelligence shapes health communication	Oral presentation
Group 4	What are the rights of nurses and how they face fake news?	Nursing ethics and quackery	To know the professional deontology of nurses and how fake news or quackery may impact their clinical practice	Oral presentation
Group 5	What are the duties of nurses toward colleagues and patients?	Responsibilities of the nurse toward colleagues and patients	To investigate the rights and responsibilities of nurses toward their colleagues and clients	Oral presentation
Group 6	How do nurses deal with psychological issues displayed by themselves, their colleagues, and clients?	Psychological problems and the potential role of nurses	To share information about psychological symptoms and prevention of burnout among HCWs	Oral presentation
Group 7	How do nurses provide palliative care?	The role of nurses in palliative care	To understand the palliative care and roles of nurses in providing care to their patients in the final stages of diseases	Oral presentation/video
Group 8	What is the Human Rights Declaration? How could it be used in nursing and health sciences?	The Human Rights Declaration	To know different rights as stated by the International Human Rights Declaration and how it should be implemented in nursing practice	Oral presentation

PBL activities were designed to align with the learning objectives of the course. The course was delivered face-to-face, with synchronous activities divided into lectures and dialogues, round tables, and tutorial meetings. Asynchronous activities supported and complemented the course content, providing additional time for project construction using the Moodle® platform (Moodle Pty Ltd, Barcelona, Spain), which provided a structured platform for project assignment and group collaboration. Students accessed the essential materials of the course and submitted project deliverables in PPT format. Regular monitoring from instructors and group leaders was provided via this platform to guide students throughout PBL.

The evaluation method was based on a formal questionnaire in which students were requested to evaluate the course. Three experts reviewed it for content validity. Table 2 shows the steps for implementing PBL in psychology and sociology of health courses.

**Table 2:** Steps in implementing a PBL tutorial PBL: project-based learning

S. no.	Steps
1.	Selecting and identifying relevant projects, as well as articulating the learning objectives
2.	Assigning projects to students and creating collaborative as well as transdisciplinary groups
3.	Providing guidance, support, and materials
4.	Facilitating group discussions
5.	Providing feedback and assessing student learning outcomes
6.	Presentation of the project

## Results

Thirty-one undergraduate students enrolled in bachelor's degree programs in Nursing, Rehabilitation (including Speech and Language Pathology, and Psychomotor Therapy), and Health Sciences responded to the survey after the completion of the course in Psychology and Sociology. The response rate was 54.3% (n = 31/57).

**Figure 1:**
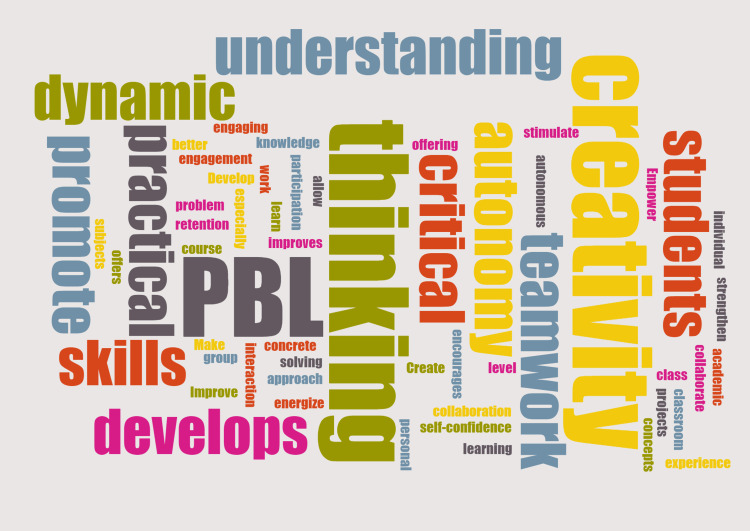
Word cloud summarizing the main self-reported skills from the PBL experience PBL: project-based learning

The outcomes of the final evaluation administered on paper indicate the success of the methodology implemented in accomplishing the intended goals. The questionnaire included two open-ended questions assessing students' perceptions of PBL and their development of critical thinking, problem-solving, teamwork, and communication skills (Figure 1).

Therefore, the students' self-reported confidence in applying learned knowledge and looking forward to using it in other courses. The outcomes of the project presented here reveal that the implementation of PBL has significantly enhanced students' perception of the value of the knowledge acquired in each project (Table 3).

**Table 3:** Responses of students to the questionnaire PBL: project-based learning

Question	Number of responses	Percentage
Following the experiment with PBL, do you feel more competent in relation to the subjects in which you attended or participated in class?	31/31	100%
Would you say that you learned more about the topics using PBL than what is generally carried out in the classroom (lectures, flipped classroom, and case study)?	17/31	54.8%
Do you believe that the development of your skills as a future nurse/health technician/rehabilitation specialist would be facilitated if PBL was used for the entire course (not just a few chapters)?	23/31	74.1%
Should we repeat this experience with other students from other faculties of medicine/dentistry/bio-engineering?	26/31	83.8%
How did your experience with PBL influence your ability to work collaboratively with your colleagues as well as your approach to problem-solving?	27/31	87%
To what extent has PBL helped you develop critical thinking skills in a positive way?	25/31	80.6%

Specifically, the survey included a question asking, "following the experiment with PBL, do you feel more competent in relation to the subjects in which you attended or participated in class?" A total of 31 students participated in the survey, and all of them reported being completely satisfied. The second question is, “Would you say that you learned more about the topics using PBL than what is generally carried out in the classroom (lectures, flipped classroom, and case study)?” A total of 54% reported learning much more with PBL in comparison with other learning methods. Additionally, 74% of students believed that PBL should be implemented in the entire course of psychology and sociology of health. With regard to their intention to replicate this experience with other students from other faculties of medicine/dentistry, 83% of respondents expressed a positive attitude. These results demonstrate that the respondents showed increased satisfaction after implementing PBL. However, it is recommended that undergraduate students from all health sciences disciplines should be enrolled in such intercultural and interdisciplinary PBL classes to develop their soft skills as well as their critical reasoning abilities.

## Discussion

The course was conducted face-to-face on campus, enabling students and professors to engage in interactive teaching and learning experiences using PBL, which is a collaborative challenge in creating an educational scenario that fosters interaction and promotes individual and collective actions. This scenario is facilitated by sharing experiences and enhancing professional understandings through collective actions between professors and students. Several authors have highlighted successful experiences in the educational process where meaningful learning is promoted through PBL as it provides contextually relevant, real-life problem-solving and proposes solutions that address these needs. A study employing an integrative literature review investigated the digital technologies used in nursing education for non-in-person teaching, such as the widely used Moodle® e-learning platform [19]. This environment enables the use of various tools like forums, chats, questionnaires, wiki-like texts, portfolios, and other possibilities, offering flexibility in terms of the content being taught.

PBL is a valuable approach in nursing education as it enables students to apply theoretical concepts to real-world situations, enhancing their understanding and critical thinking skills [20]. It also encourages collaboration among peers, enhancing communication and teamwork skills essential in a profession that often involves interdisciplinary collaboration [21]. The emphasis on problem-solving and decision-making in a simulated environment helps students build confidence and competence, contributing to their overall professional development [22]. Given the dynamic and ever-changing nature of healthcare, integrating PBL into nursing education ensures that students actively learn how to navigate the complexities of patient care, fostering a deeper and more meaningful understanding of the concepts that will serve them well throughout their nursing careers. PBL also provides opportunities for students to work on projects that require input from multiple perspectives, preparing them for the interdisciplinary nature of healthcare. It fosters effective communication, teamwork, and the ability to navigate the complexities of patient care in a collaborative environment. In real-world healthcare settings, successful patient outcomes often depend on seamless collaboration among healthcare professionals, making the development of these skills crucial for nursing students [23].

According to the study by Stirling [24], a low alignment was found between faculty teaching preferences and student learning styles, with only about 20% alignment. However, course grades were discovered to be related to both learning styles and teaching preferences. Students with specific learning styles, such as Sensing-Thinking, achieved significantly higher scores than those with other learning styles. Moreover, students instructed by faculty members with an Intuitive-Feeling teaching preference scored significantly higher than those taught by faculty with other teaching preferences. These results imply that nursing educators must consider and accommodate various learning styles to offer effective instruction and improve student outcomes. Similar to the research by Katebi et al. [7], most undergraduate nursing students prefer a visual learning style, followed by auditory and tactile styles. These findings are essential for determining the efficacy of diverse training methods in enhancing nursing students' clinical competence, which is vital for their future professional practice.

PBL in health sciences learning and teaching: from hard to soft, life, and self-skills

PBL has been increasingly recognized as a valuable educational approach in health sciences, with a focus on developing not only hard skills but also soft, life, and self-skills. Ruan et al. [25] demonstrate that experiential learning, which is closely related to PBL, can effectively improve nursing soft skills, suggesting its suitability for nursing education. Similarly, Shen et al. [26] provide evidence that individual factors and PBL can positively influence the development of nontechnical skills, such as conflict resolution, which are crucial in nursing practice.

Developing skills and competencies has been a focal point in various higher education disciplines for several decades [16,27]. Students are expected to develop essential competencies and skills with a solid theoretical and practical foundation [17]. These skills are embedded in clinical practice through a range of verbal and nonverbal attitudes, appropriate conduct, and behavior that adapts to each practical situation [17]. Classical models for developing hard skills (theoretical and academic knowledge) and soft skills (inter-/intrapersonal traits and attitudes) have been prevalent in fields like marketing, engineering, technology, and industry, where technical knowledge is required [26]. The need to develop soft skills arose from the pressing need to create a successful model of learning, practice, and employability that combined core technical, professional, and attitudinal or communicative qualities and skills. Communication is conceptualized differently in these areas, whereas in nursing education, it is considered the cornerstone and prerequisite for admission, academic, and professional success [17]. A new conceptual framework for learning in nursing and allied health sciences should focus on fostering both theoretical clinical skills (hard skills) and inter- and intrapersonal attitudes (soft skills). Self- and life skills are essentially those abilities that help enhance mental well-being. They could be an interesting area to investigate to bridge the gap between hard and soft skills [26].

In a recent meta-analysis conducted by Wijnia et al. [28], the impact of the student-centered problem and PBL approaches on student motivation has been compared to traditional teacher-centered and lecture-based methods. The authors conceptualized motivation as a complex construct that includes students' beliefs about their competence and control, their perceptions of the value of tasks in terms of interest and importance, and their motivations for engaging in tasks, whether intrinsic or extrinsic. No significant differences in motivation were observed among the three instructional methods; however, the effects were more substantial when PBL was implemented within a single course rather than across a curriculum. Furthermore, larger effects were noted in specific academic fields, particularly in healthcare and science, technology, engineering, and mathematics (STEM), compared to others. While the overall influence of PBL on motivation is favorable, the complex interactions among factors such as academic discipline and implementation strategy highlight the necessity for a tailored approach to effectively utilize these instructional methods to enhance student motivation.

Limitations of the study

PBL has shown promise in psychology and sociology courses, fostering active student engagement, teamwork, and exploring various themes. However, since the faculty of nursing and health sciences has just started in 2023-2024, the students enrolled in this course are among the first cohort in health sciences. This limits, at the moment, the representativeness of the broader population of undergraduate nursing and health sciences students. Yet, more longitudinal and follow-up studies are necessary to verify these positive outcomes and to extend them to other courses and disciplines. Furthermore, evaluating students' critical thinking skills and perceptions of PBL is reported based on a single questionnaire. However, critical thinking, especially in the context of PBL, is a complex construct that may be difficult to assess. Therefore, future studies should use tools that are well-established and available for assessing critical thinking more comprehensively, such as the California Critical Thinking Skills Test or other validated critical thinking scales. Incorporating such psychometric instruments would provide a more accurate and robust assessment of how PBL has influenced students' critical thinking development.

## Conclusions

Implementing PBL in the health sciences course proved effective in achieving educational objectives, particularly in fostering skills required for planning, constructing, developing, and assessing health-related projects. Mentorship using PBL played a vital role in the students' growth and productivity, allowing them autonomy and freedom to explore and learn about health psychology and sociology, as showed by their satisfaction in the course evaluation. Furthermore, PBL promotes student collaboration, motivation and engagement by imparting a sense of purpose and relevance to their learning.

## References

[1] Almulla MA (2020). The effectiveness of the project-based learning (PBL) approach as a way to engage students in learning. Sage Open.

[2] Almaguer I, Diaz Z, Esquierdo JJ (2015). Project-based learning: innovative pedagogy for 21st-century English learners. Teach Educ Pract.

[3] Warr M, West RE (2020). Bridging academic disciplines with interdisciplinary project-based learning: challenges and opportunities. Interdiscip J Probl Based Learn.

[4] Chang TS, Wang HC, Haynes AM, Song MM, Lai SY, Hsieh SH (2022). Enhancing student creativity through an interdisciplinary, project-oriented problem-based learning undergraduate curriculum. Think Skills Creat.

[5] Hwang RH, Hsiung PA, Chen YJ, Lai CF (2017). Innovative project-based learning. Emerging Technologies for Education.

[6] Sheng T, Hu Q (2012). Human extracellular superoxide dismutase recombination: a project-based learning program in biochemistry designed for nursing students. Asian J Nurs Educ Res.

[7] Katebi MS, Ahmadi AA, Jahani H, Mohalli F, Rahimi M, Jafari F (2020). The effect of portfolio training and clinical evaluation method on the clinical competence of nursing students. J Nurs Midwifery Sci.

[8] Assadi HS, Shariati AA, Haghighi SH, Latifi SM, Sheini JP (2014). Effects of clinical education and evaluation with portfolio method on nursing students' satisfaction. J Clin Nurs Midwifery.

[9] Tsai PR, Lee TT, Lin HR, Lee-Hsieh J, Mills ME (2015). Nurses' perceptions of e-portfolio use for on-the-job training in Taiwan. Comput Inform Nurs.

[10] Pascon DM, Vaz DR, Peres HH, Leonello VM (2022). Project-based learning in remote teaching for undergraduate nursing students. Rev Esc Enferm USP.

[11] Khatiban M, Sangestani G (2014). The effects of using problem-based learning in the clinical nursing education on the students' outcomes in Iran: a quasi-experimental study. Nurse Educ Pract.

[12] Khalili R, Khaghanizade M, Sirati Nir M, Mokhtari Noori J, Zicker F (2015). Evidence-based nursing education: a scoping review. Int J Med Rev.

[13] Kokotsaki D, Menzies V, Wiggins A (2016). Project-based learning: a review of the literature. Improv Sch.

[14] Egenrieder JA (2010). Facilitating student autonomy in project-based learning to foster interest and resilience in STEM education and STEM careers. J Wash Acad Sci.

[15] Stolk JD, Martello R (2015). Can disciplinary integration promote students’ lifelong learning attitudes and skills in project-based engineering courses?. Int J Eng Educ.

[16] Taiebine M, Keegan LC (2022). E-mentorship in speech-language pathology. Teach Learn Commun Sci Disord.

[17] Kim Y (2021). Effect of projet-based learning on the creative personality, teamwork competence and self-regulated efficacy of undergraduate nursing students. Turk J Comput Math Educ.

[18] Larson LC (2007). Supervision: student clinicians develop independence by assessing their own clinical skills. Perspect Adm Supervision.

[19] Amandu GM, Muliira JK, Fronda DC (2013). Using Moodle e-learning platform to foster student self-directed learning: experiences with utilization of the software in undergraduate nursing courses in a Middle Eastern university. Procedia Soc Behav Sci.

[20] Boss S, Krauss J (2022). Reinventing Project-Based Learning: Your Field Guide to Real-World Projects in the Digital Age. Int Soc Technol Educ.

[21] Rohm AJ, Stefl M, Ward N (2021). Future proof and real-world ready: the role of live project-based learning in students’ skill development. J Mark Educ.

[22] Dehdashti A, Mehralizadeh S, Kashani MM (2013). Incorporation of project-based learning into an occupational health course. J Occup Health.

[23] Nagarajan S, Overton T (2019). Promoting systems thinking using project- and problem-based learning. J Chem Educ.

[24] Stirling BV (2017). Results of a study assessing teaching methods of faculty after measuring student learning style preference. Nurse Educ Today.

[25] Ruan X, Lu Y, Li H, Qiong X, Xu D (2019). Application of experiential learning in nursing soft skills training. Chin J Pract Nurs.

[26] Shen Y, Mu S, Tang W, Wang X (2016). Influence of nursing students’ personal factors on the teaching effect of nurse patient communication experience. Chin J Med Educ Res.

[27] Taiebine M (2023). E-learning and intercultural online education pre-and post-Covid-19. Transforming Teaching and Learning Experiences for Helping Professions in Higher Education.

[28] Wijnia L, Noordzij G, Arends LR, Rikers RM, Loyens SM (2024). The effects of problem-based, project-based, and case-based learning on students’ motivation: a meta-analysis. Educ Psychol Rev.

